# Supplementary material for: Modelling spatiotemporal trends in the frequency of genetic mutations conferring insecticide target-site resistance in African mosquito malaria vector species
Source: BMC Biol. 2022 Feb 15;20:46. doi: 10.1186/s12915-022-01242-1 (PMC8845222; doi:10.1186/s12915-022-01242-1)
Supplement: Supplementary file 1 — Additional file 1: Additional results and information to support this manuscript, including: Figure S1. Results of 10-fold out-of-sample posterior validation for the spatiotemporal model ensemble. Figures S2 & S3. Sampling locations of the observed frequencies of the Vgsc-995F and Vgsc-995S markers in Africa that were included in our modelling analysis. Figures S4 & S5. The number of samples of the frequencies of the Vgsc-995F and Vgsc-995S markers included in our data set for each country. Figure S6. The total number of Vgsc allele frequency observations that were used to inform the spatiotemporal model ensemble by each year and each vector species. Figure S7. The abundance of An. arabiensis relative to the combined abundance of An. gambiae and An. coluzzii in the nine mapped countries. Figures S8, S9, S10 & S11. Associations between predicted frequencies of Vgsc mutations across species. Figures S12, S13 & S14. The ITN coverage across the nine mapped countries in years 2005, 2011 and 2017. Figure S15. The predicted L1014F frequency in the An. gambiae complex derived from combining species-specific frequencies (eq 1) vs observed frequencies. Table S1. The root mean square error (RMSE) and the mean absolute error (MAE) across the out-of-sample predictions of all Vgsc allele frequency observations, obtained using 10-fold cross validation. Table S2. Countries that were included in (i) each type of mapping analysis, and (ii) in the analysis of relationships between deltamethrin resistance phenotype and Vgsc mutation frequencies. Table S3. Descriptions of each potential explanatory variable used in the ensemble model. [file 12915_2022_1242_MOESM1_ESM.pdf]

SUPPLEMENTARY MATERIAL

"Modelling spatiotemporal trends in the frequency of genetic mutations conferring insecticide target-site resistance in African malaria vector species"

Penelope A. Hancock, Amy Lynd, Antoinette Wiebe, Maria Devine, John Essandoh, Francis Wat'senga, Emile Z. Manzambi, Fiacre Agossa, Martin J. Donnelly, David Weetman, Catherine L. Moyes

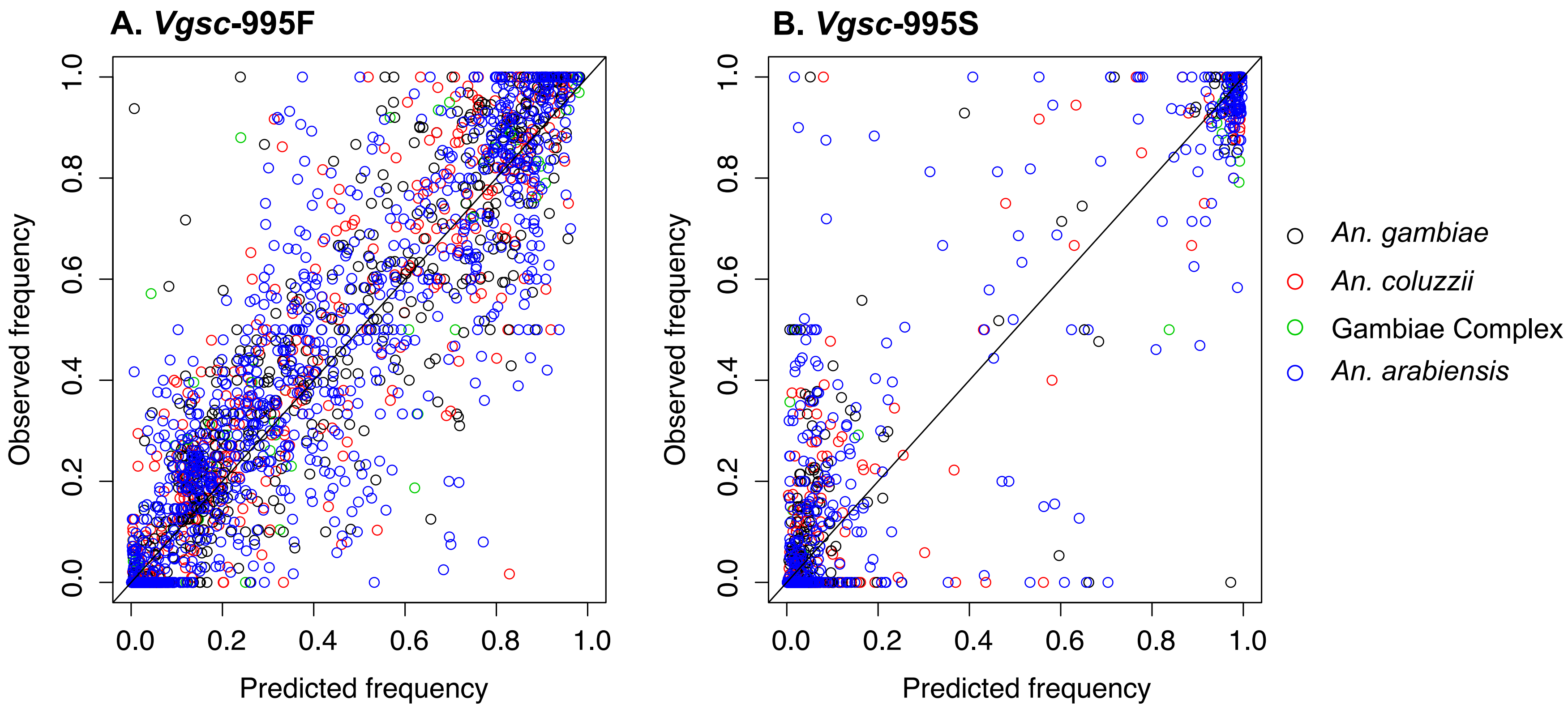

Figure S1. Results of 10-fold out-of-sample posterior validation for the spatiotemporal model ensemble. The horizontal and vertical axes show model-predicted and observed frequencies of (A) *Vgsc*-995F and (B) *Vgsc*-995S across the four different vector species categories: *An. gambiae* (black markers), *An. coluzzii* (red markers), *An. gambiae s.l.* (green markers), and *An. arabiensis* (blue markers).

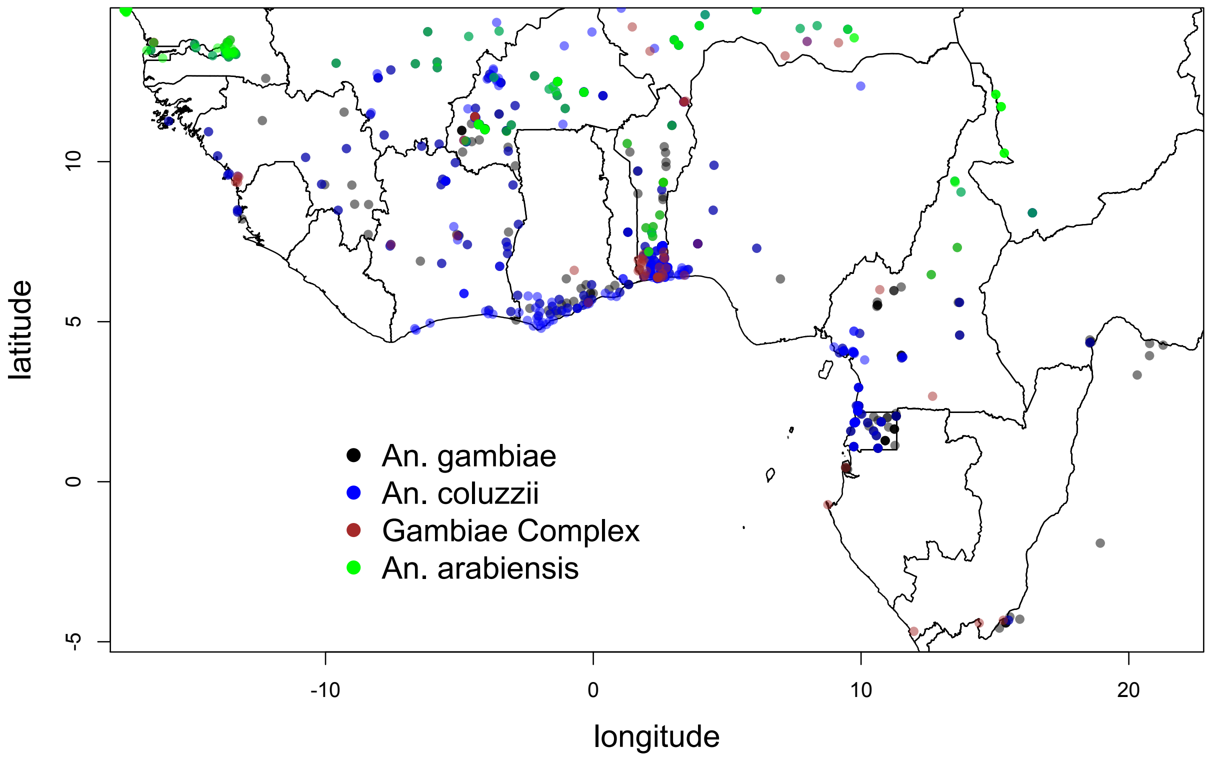

Figure S2. Sampling locations of the observed frequencies of the *Vgsc*-995F and *Vgsc*-995S markers in the western region of Africa that were included in our modelling analysis.

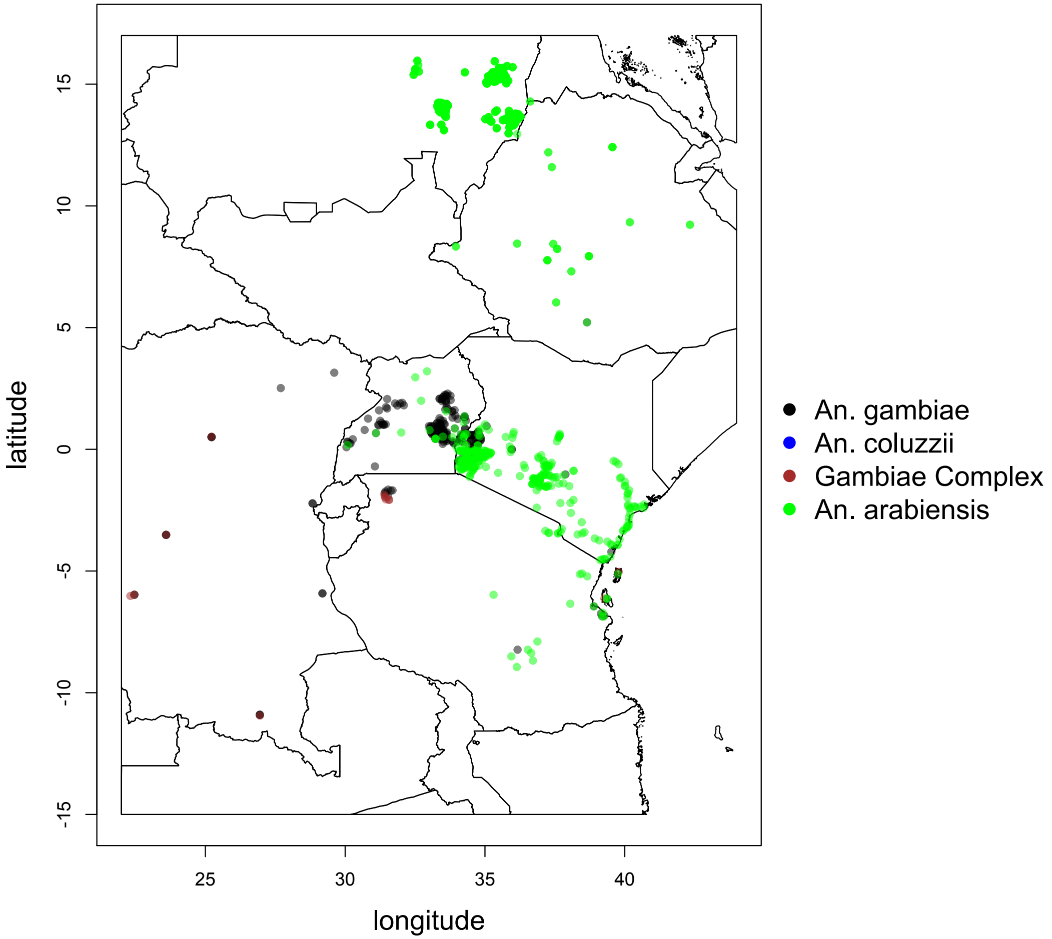

Figure S3. Sampling locations of the observed frequencies of the *Vgsc*-995F and *Vgsc*-995S markers in the eastern region of Africa that were included in our modellng analysis.

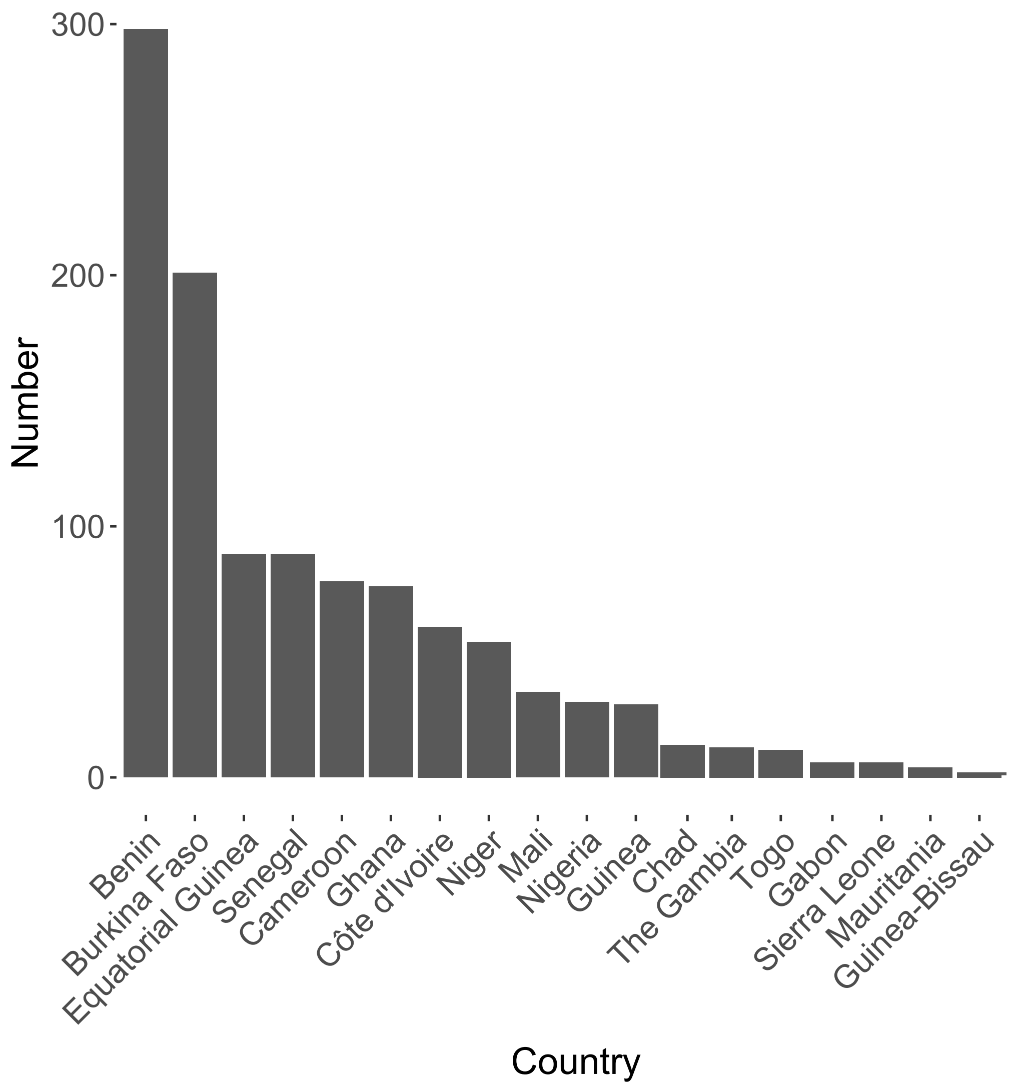

Figure S4. The number of samples of the frequencies of the *Vgsc*-995F and *Vgsc*-995S markers included in our data set for each country across west Africa.

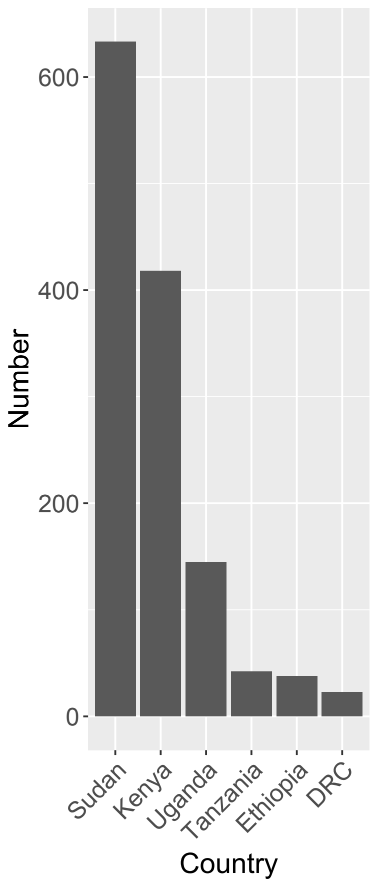

Figure S5. The number of samples of the frequencies of the *Vgsc*-995F and *Vgsc*-995S markers included in our data set for each country across east Africa.

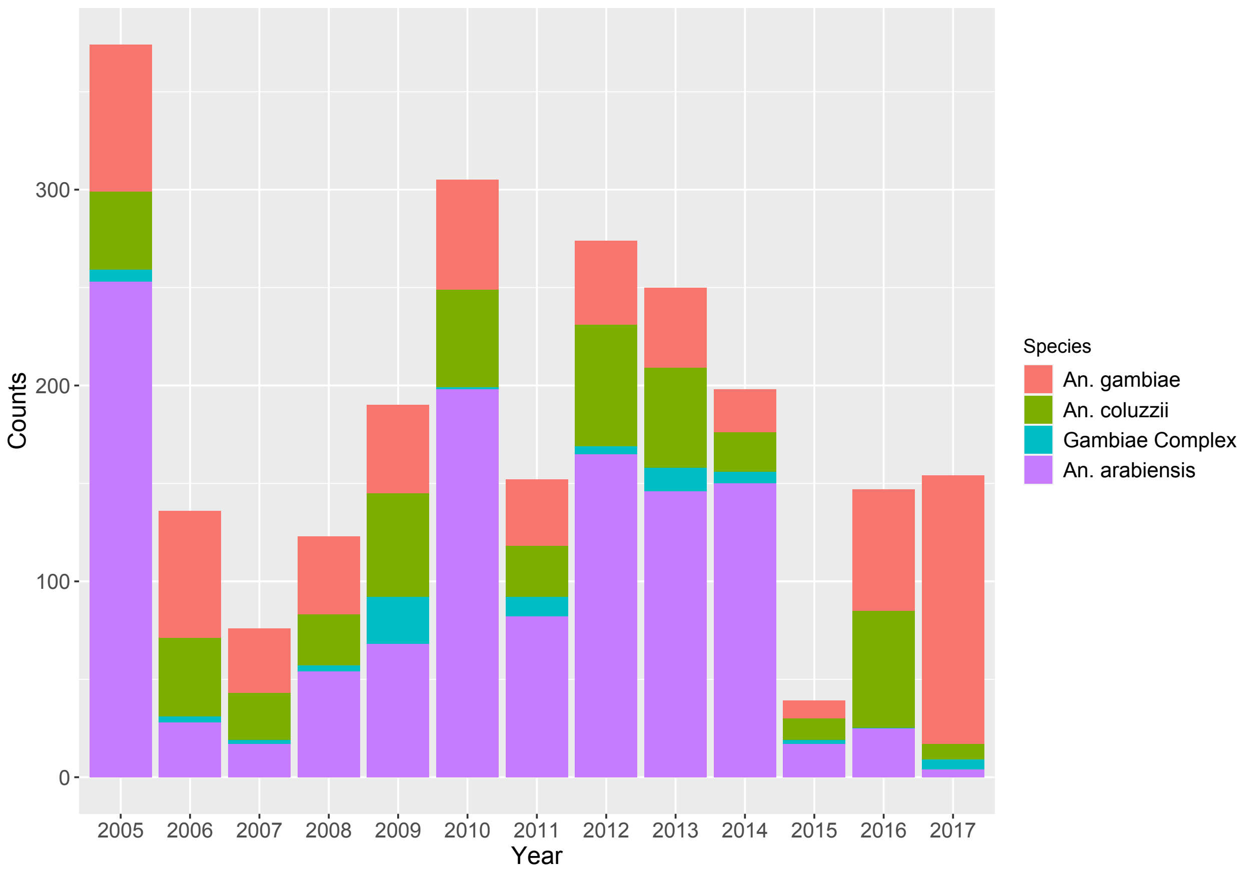

Figure S6. The total number of *Vgsc* allele frequency observations that were used to inform the spatiotemporal model ensemble by each year and each vector species.

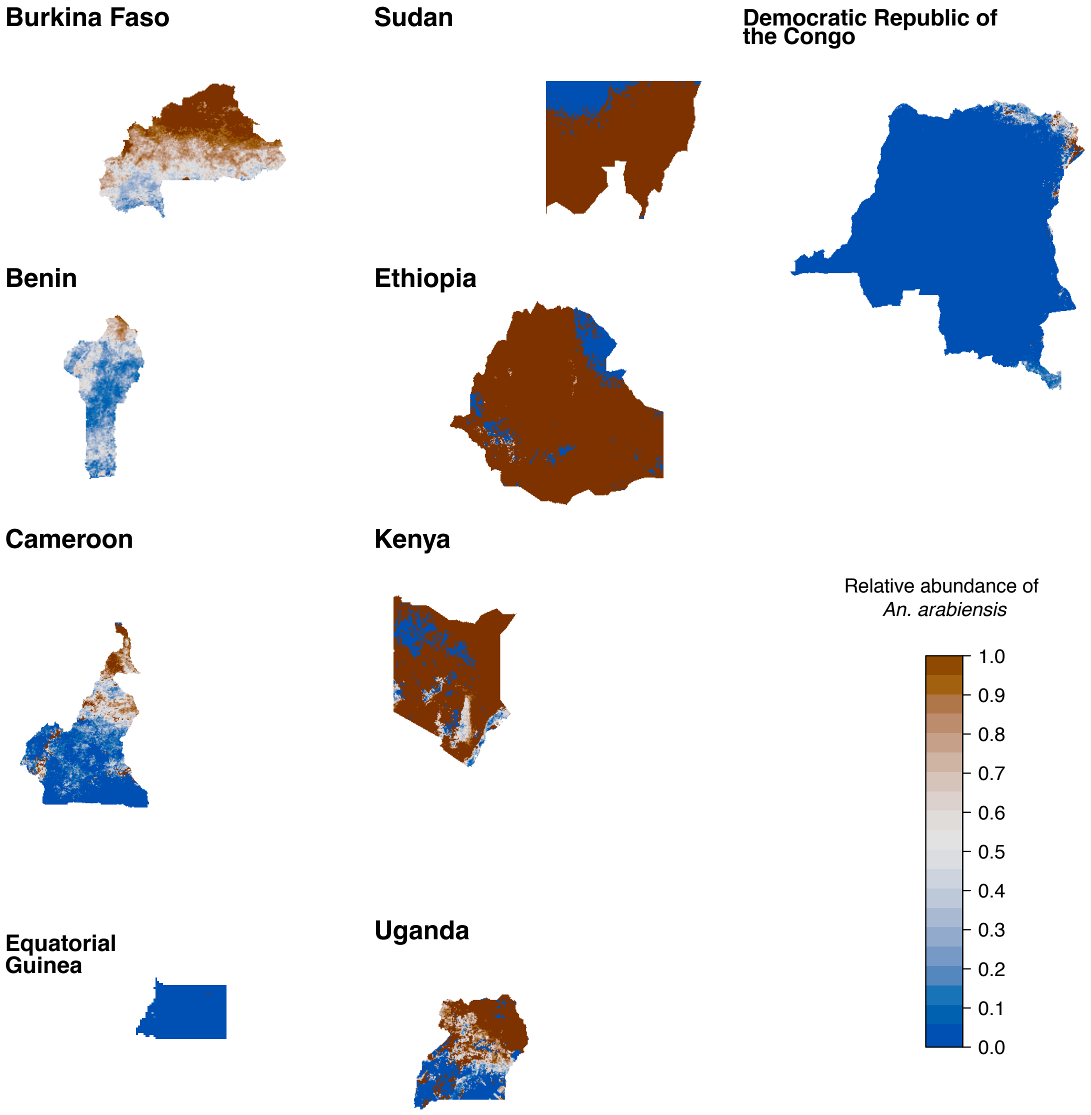

Figure S7. The abundance of *An. arabiensis* relative to the combined abundance of *An. gambiae* and *An. coluzzii* in the nine mapped countries. Western countries are shown in the first column from the left, eastern countries are shown in the second column from the left, and central African countries are shown in the third column from the left.

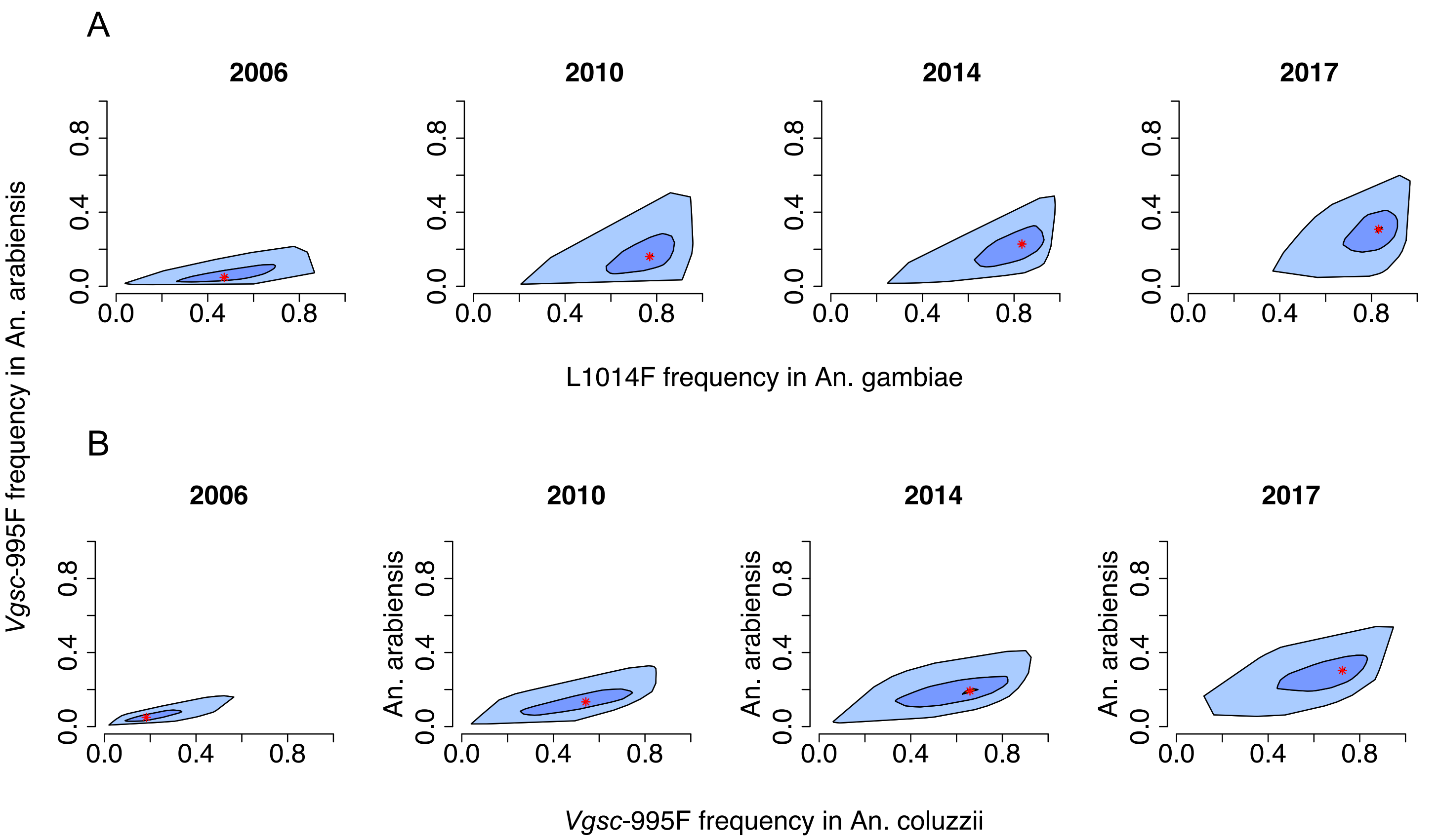

Figure S8. Associations between the predicted frequency of the *Vgsc*-995F allele in **A.** *An. gambiae* and *An. arabiensis*; **B.** *An. coluzzii* and *An. arabiensis.* Bagplots show the distribution across all mapped pixels within four countries in west Africa: Burkina Faso, Benin, Cameroon and Equatorial Guinea. The red asterisk shows the median, the dark blue shaded area contains 50% of all data points and the line blue shaded area contains all data points. Plots for four years are shown (from left to right): 2006, 2010, 2014 and 2017. The Pearson correlation coefficient between these predicted Vgsc-995F frequencies in *An. gambiae* and *An. arabiensis* for the years 2006, 2010, 2014 and 2017 are *r*=0.77 (CI=0.76,0.78), *r*=0.59 (CI=0.58,0.6), *r*=0.73 (CI=0.72,0.74), *r*=0.48 (CI=0.46,0.49). The Pearson correlation coefficient between these predicted *Vgsc*-995F frequencies in *An. coluzzii* and *An. arabiensis* for the years 2006, 2010, 2014 and 2017 are *r*=0.78 (CI=0.77,0.79), *r*=0.82(CI=0.81,0.83), *r*=0.74 (CI=0.73,0.75), *r*=0.66 (CI=0.65,0.67).Credible intervals were determined by boostrapping using the R package “boot”.

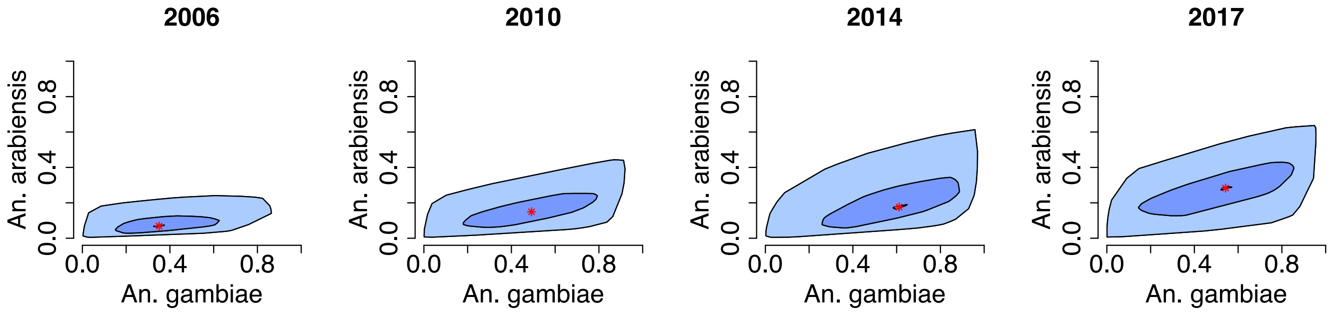

Figure S9. Associations between the predicted frequency of the *Vgsc*-995F allele in *An. gambiae* and *An. arabiensis.* Bagplots show the distribution across all mapped pixels within four countries in east Africa: Ethiopia, Sudan, Uganda and Kenya. The red asterisk shows the median, the dark blue shaded area contains 50% of all data points and the line blue shaded area contains all data points. Plots for four years are shown (from left to right): 2006, 2010, 2014 and 2017. The Pearson correlation coefficient between these predicted *Vgsc*-995F frequencies in *An. gambiae* and *An. arabiensis* for the years 2006, 2010, 2014 and 2017 are *r*=0.69 (CI=0.68,0.69), *r*=0.69 (CI=0.68,0.69), *r*=0.72 (CI=0.71,0.72), *r*=0.59 (CI=0.58,0.6). Credible intervals were determined by boostrapping using the R package "boot".

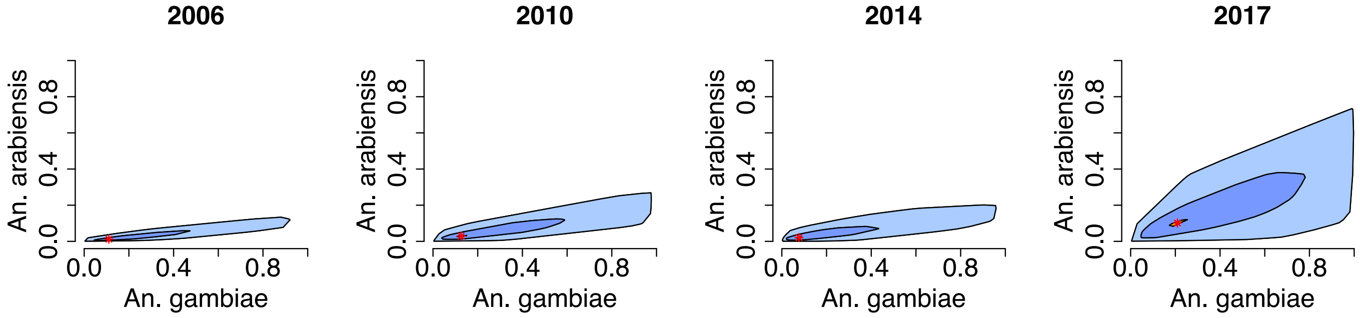

Figure S10. Associations between the predicted frequency of the *Vgsc*-995S allele in *An. gambiae* and *An. arabiensis.* Bagplots show the distribution across all mapped pixels within four countries in east Africa: Ethiopia, Sudan, Uganda and Kenya. The red asterisk shows the median, the dark blue shaded area contains 50% of all data points and the line blue shaded area contains all data points. Plots for four years are shown (from left to right): 2006, 2010, 2014 and 2017. The Pearson correlation coefficient between these predicted *Vgsc*-995F frequencies in *An. gambiae* and *An. arabiensis* for the years 2006, 2010, 2014 and 2017 are *r*=0.61 (CI=0.6,0.62), *r*=0.7 (CI=0.7,0.71), *r*=0.64 (CI=0.63,0.65), *r*=0.76 (CI=0.75,0.76). Credible intervals were determined by boostrapping using the R package "boot".

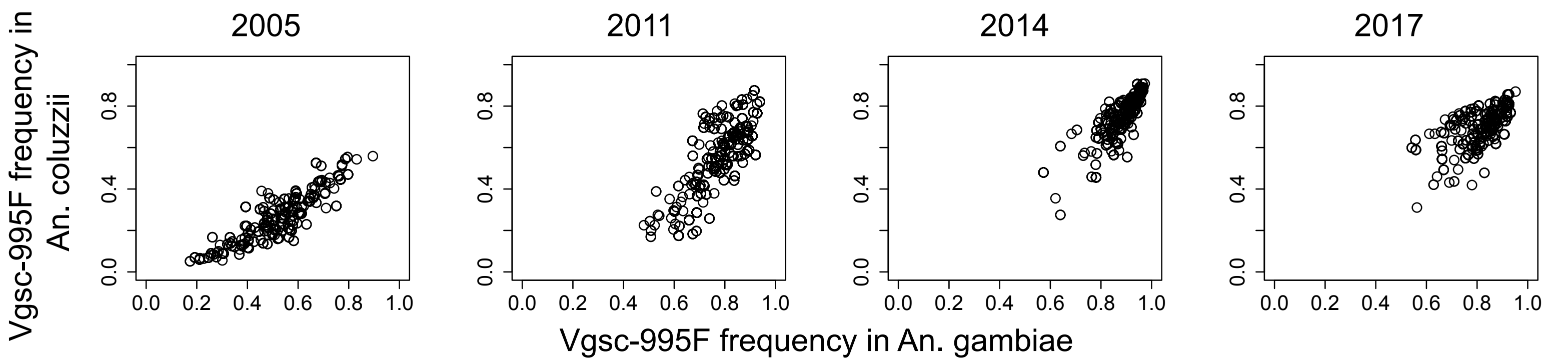

Figure S11. Associations between the predicted frequency of the *Vgsc*-995F allele in *An. gambiae* and *An. coluzzii* across sampling locations. Scatterplots show predicted frequencies at the subset of spatial locations where observed *Vgsc*-995F allele frequencies are available within four countries in west Africa: Burkina Faso, Benin, Cameroon and Equatorial Guinea. Plots for four years are shown (from left to right): 2006, 2010, 2014 and 2017.

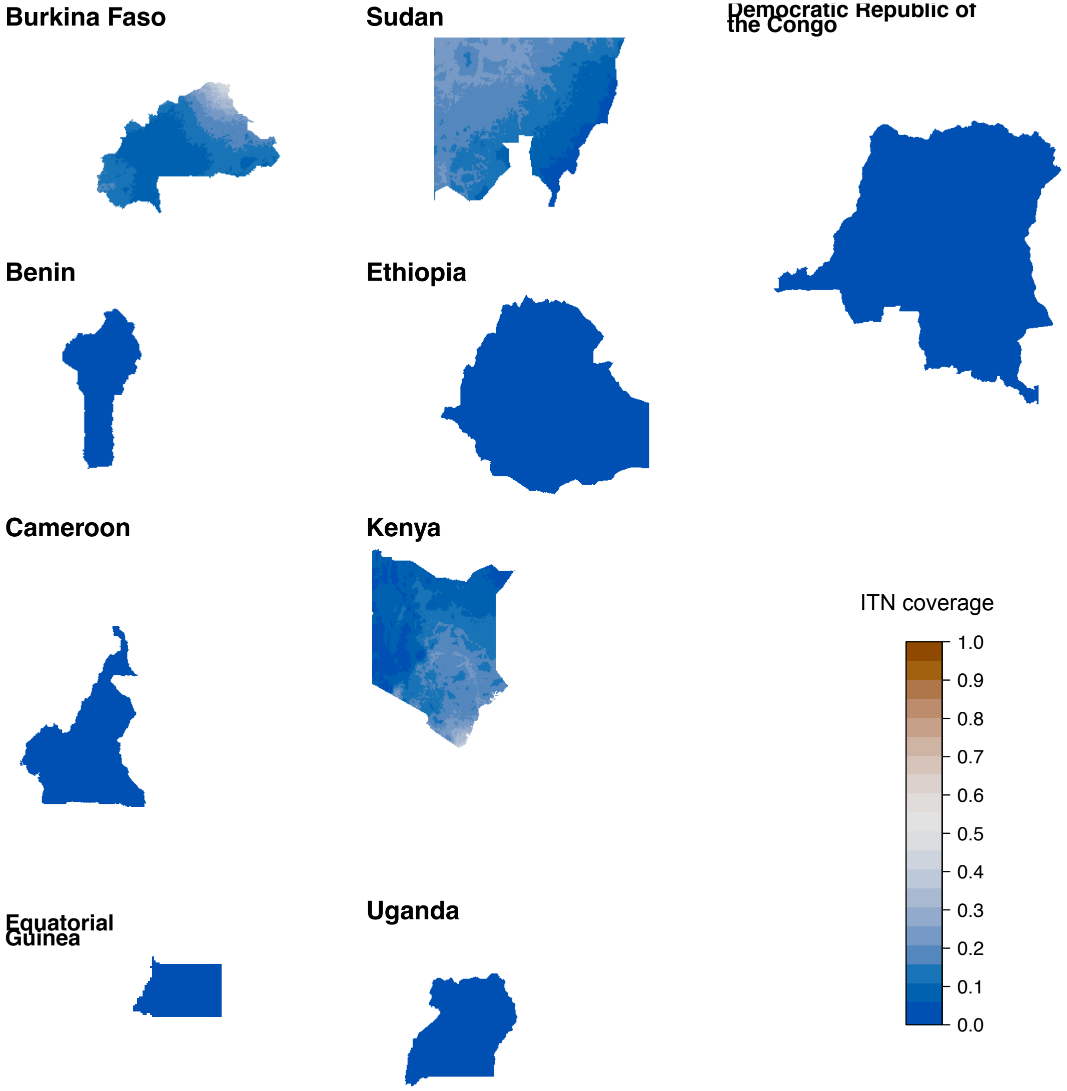

Figure S12. The ITN coverage in 2005 in the nine mapped countries. Western countries are shown in the first column from the left, eastern countries are shown in the second column from the left, and central African countries are shown in the third column from the left.

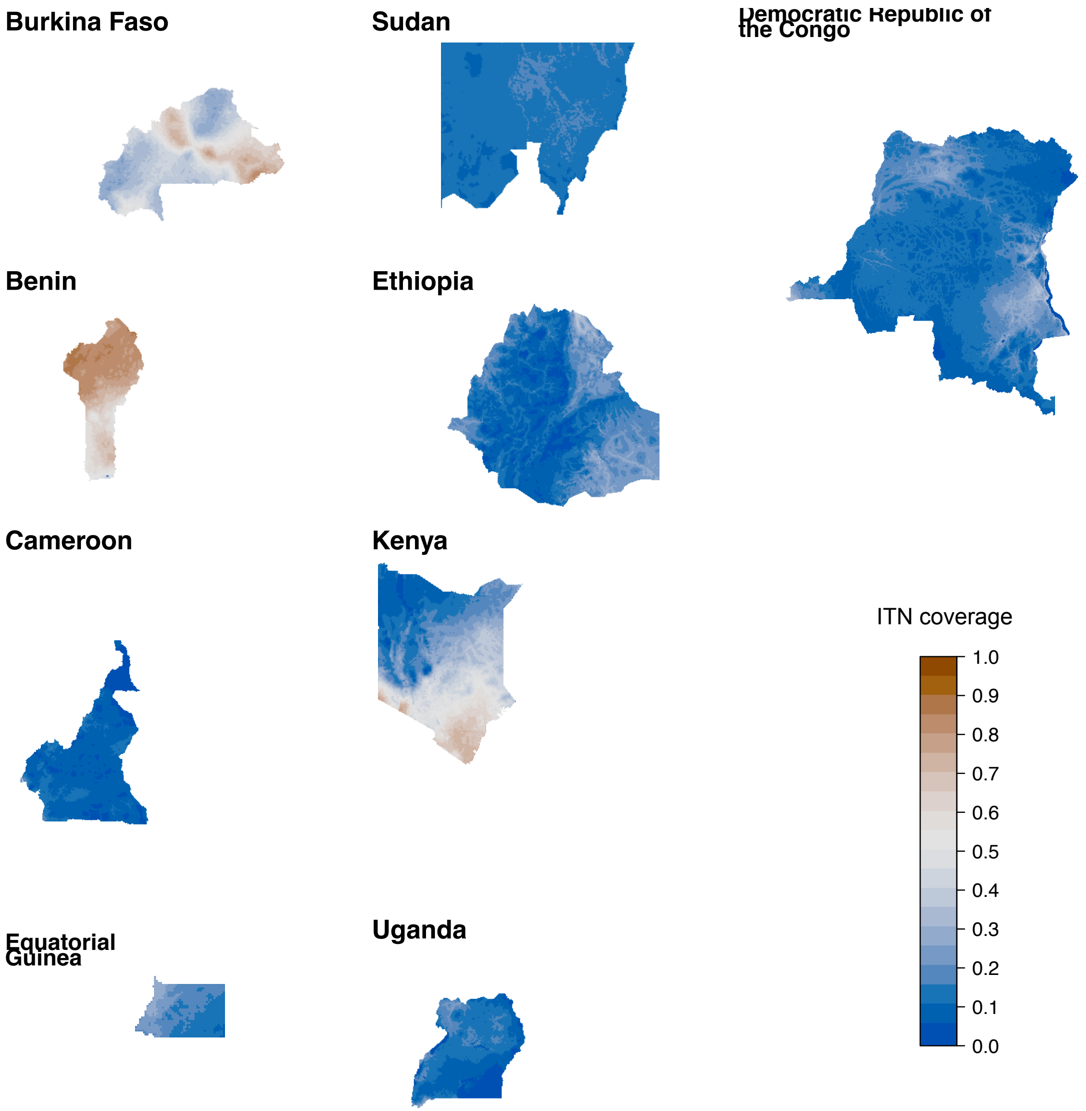

Figure S13. The ITN coverage in 2011 in the nine mapped countries. Western countries are shown in the first column from the left, eastern countries are shown in the second column from the left, and central African countries are shown in the third column from the left.

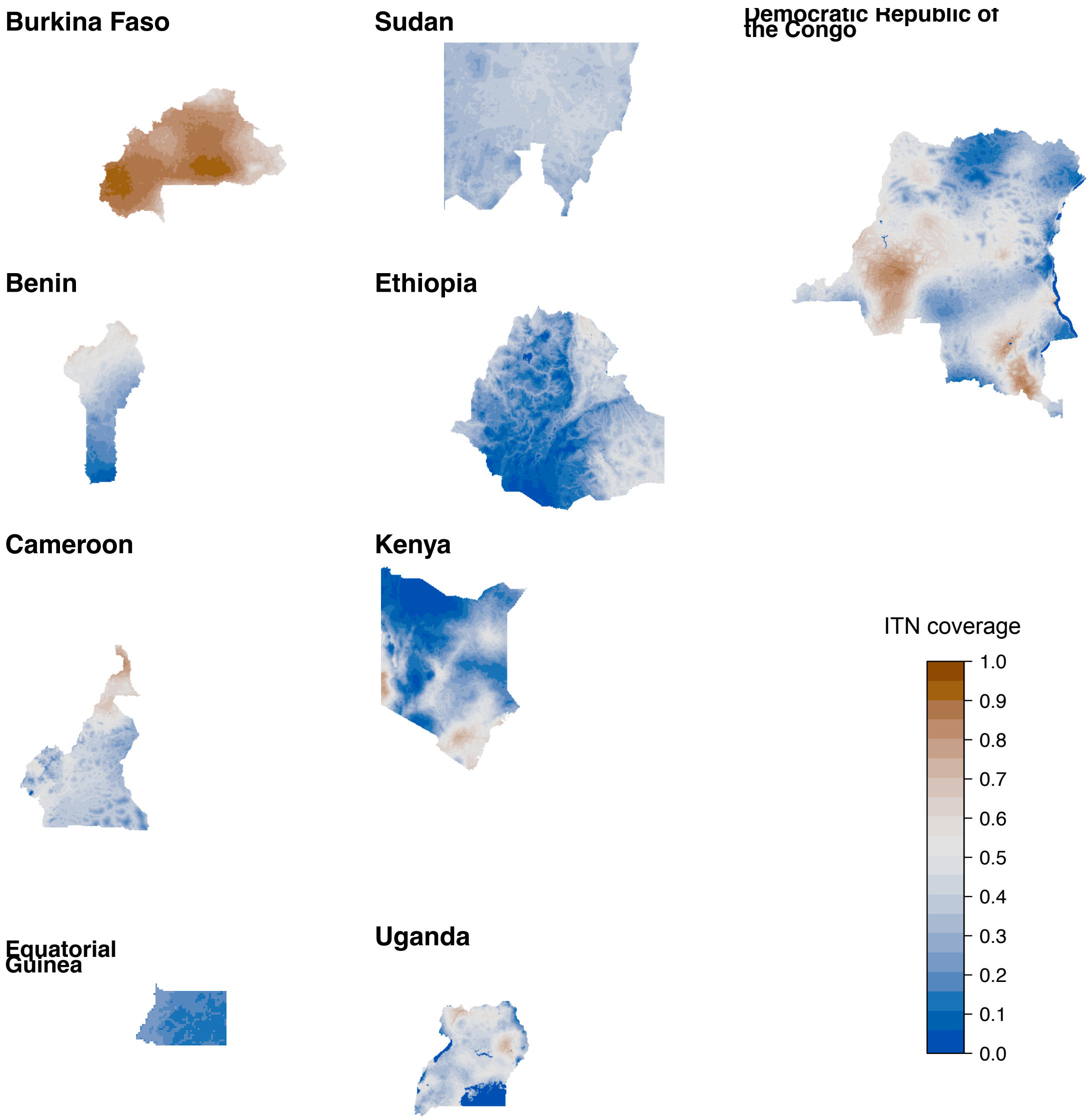

Figure S14. The ITN coverage in 2017 in the nine mapped countries. Western countries are shown in the first column from the left, eastern countries are shown in the second column from the left, and central African countries are shown in the third column from the left.

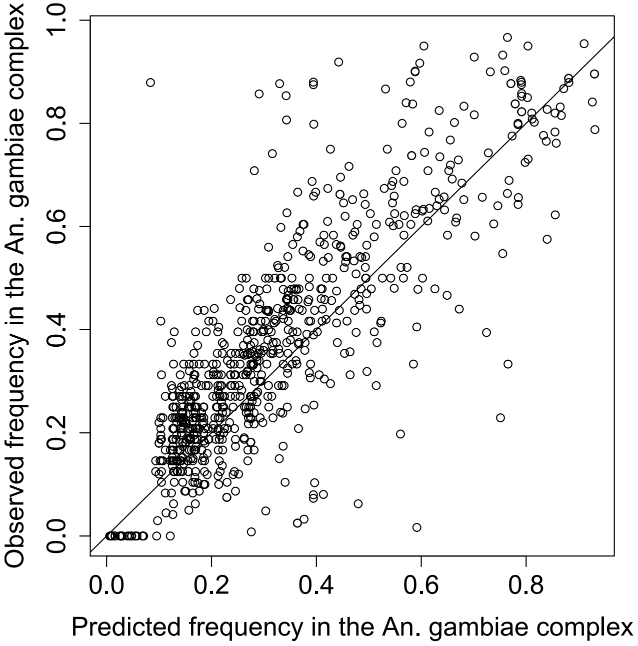

Figure S15. The predicted L1014F frequency in the *An. gambiae* complex derived from combining species-specific frequencies (eq 1) vs observed frequencies.

Table S1. The root mean square error (RMSE) and the mean absolute error (MAE) across the out-of-sample predictions of all *Vgsc* allele frequency observations, obtained using 10-fold cross validation. The RMSE and MAE are calculated across all observed frequencies of *Vgsc*-995L, *Vgsc*-995S and *Vgsc*-995F alleles. The out-of-sample RMSE and MAE for the multinomial logit model ensemble, together with that of each individual model constituent (XGB, RF and NN), is shown.

Model	Out-of-sample RMSE	Out-of-sample MAE
Multinomial logit meta-model	0.137	0.083
XGB	0.142	0.091
RF	0.145	0.10
NN	0.155	0.10

Table S2. Countries that were included in (i) each type of mapping analysis, according to mosquito species and the type of *Vgsc* mutation mapped, and (ii) in the analysis of relationships between deltamethrin resistance phenotype and *Vgsc* mutation frequencies. For countries that were not included, the reason for exclusion is provided.

	Mapping of ***Vgsc*-995F** frequencies in ***An. gambiae***	Mapping of ***Vgsc*-995F** frequencies in ***An. coluzzii***	Mapping of ***Vgsc*-995F** frequencies in ***An. arabiensis***	Mapping of ***Vgsc*-995S** frequencies in ***An. gambiae***	Mapping of ***Vgsc*-995S** frequencies in ***An. coluzzii***	Mapping of ***Vgsc*-995S** frequencies in ***An. arabiensis***	Analysis of association between mapped *Vgsc*-995F frequencies and the prevalence of mortality to deltamethrin
Burkina Faso	Yes	Yes	Yes	No. The frequency of *Vgsc*-995S is very low in west Africa (observed frequencies were zero in ~80% of the samples from the west African countries).	No. Insufficient number of samples where *Vgsc*-995S has been found in *An. coluzzii.*	No. The frequency of *Vgsc*-995S is very low in west Africa.	Yes
Benin	Yes	Yes	Yes	No. Same reason as for Burkina Faso.	No. Same reason as for Burkina Faso.	No. Same reason as for Burkina Faso.	Yes
Cameroon	Yes	Yes	Yes	No. Same reason as for Burkina Faso.	No. Same reason as for Burkina Faso.	No. Same reason as for Burkina Faso.	Yes
Equatorial Guinea	Yes	Yes	Yes	No. Same reason as	No. Same reason as	No. Same reason as	No. Very few deltamethrin

				for Burkina Faso.	for Burkina Faso.	for Burkina Faso.	bioassay records are available for Equatorial Guinea.
Democratic Republic of the Congo	Yes	No. Insufficient number of samples representing *An. coluzzii.*	No. The relative abundance of *An. arabiensis* is very low in the DRC.	No. Insufficient number of samples measuring *Vgsc*-995S frequency.	No. Same reason as for Burkina Faso.	No. The relative abundance of *An. arabiensis* is very low in the DRC.	No. Very few deltamethrin bioassay results are available for the DRC.
Sudan	Yes	No. The relative abundance of *An. coluzzii* is very low in east Africa.	Yes	Yes	No. Same reason as for Burkina Faso.	Yes	Yes
Ethiopia	Yes	No. Same reason as for Sudan.	Yes	Yes	No. Same reason as for Burkina Faso.	Yes	Yes
Kenya	Yes	No. Same reason as for Sudan.	Yes	Yes	No. Same reason as for Burkina Faso.	Yes	No. The *Vgsc*-995F frequency is very low in the Kenyan samples (see Methods).
Uganda	Yes	No. Same reason as for Sudan.	Yes			Yes	No. There were insufficient deltamethrin bioassay results for Uganda.

Table S3. Descriptions of each potential explanatory variable used in the ensemble model.

Short name	Description	Temporal resolution	Lags	URL	Date accessed	Citation
**Insecticide-based malaria intervention coverage**						
ITN coverage	ITN coverage (proportion of people protected)	annual	0, 1, 2, 3 years	https://map.ox.ac.uk/explorer/#/	n/a	39
Anopheles gambiae **complex species**						
Arabiensis vs gambiae/coluzzi i	Proportional abundance of *An. arabiensis* to *An. coluzzii/gambiae*	static	n/a	n/a	n/a	41
**Processes associated with pesticide fate in the environment**						
Leaching	Infiltration and percolation of rain or irrigation water to deeper groundwater layers.	n/a	n/a	n/a	n/a	42
Surface runoff generation	Mechanisms involved in the generation of surface runoff of rain or irrigation water.	n/a	n/a	n/a	n/a	42
Surface runoff transfer	Transfer of rain or irrigation water overland to other streams or surface water.	n/a	n/a	n/a	n/a	42
Surface runoff accumulation	Streams or surface waters where rain or irrigation water accumulates.	n/a	n/a	n/a	n/a	42
Sedimentation	Soil particles in suspension settle out of fluid, water in this instance, and come to rest.	n/a	n/a	n/a	n/a	42
Soil storage and filtering capacity	Capacity of a soil to store and filter chemical substances.	n/a	n/a	n/a	n/a	42
Volatilization†	Chemical substances convert from a liquid or solid state to a gaseous or vapour state.	monthly	n/a	n/a	n/a	42
**Crop and livestock variables**						
Cropland percentage	Proportion of the pixel area covered by annual crops (temporary crops with harvest period or bare soil)	annual	0, 1, 2, 3 years	https://modis.gsfc.nasa.gov/data/dataprod/mod12.php	30 July 2018	43
Cropland-natural vegetation percentage	Proportion of the pixel area covered by a mosaic of annual crops and natural vegetation (mosaic of cropland, forest, shrubland or grassland)	annual	0, 1, 2, 3 years	https://modis.gsfc.nasa.gov/data/dataprod/mod12.php	30 July 2018	43
Rice	Rice production in 2005 (metric tonne)	static	n/a	http://harvestchoice.org/data/rice_p	8 Feb 2018	44
Cotton	Cotton production in 2005 (metric tonne)	static	n/a	https://harvestchoice.org/data/cott_p	8 Feb 2018	45
Sugar cane	Sugar cane production in 2005 (metric tonne)	static	n/a	https://harvestchoice.org/data/sugc_p	8 Feb 2018	46

Maize	Maize production in 2005 (metric tonne)	static	n/a	https://harvestchoice.org/data/maiz_p	8 Feb 2018	47
Non-food	Non-food crop production in 2005 (metric tonne)	static	n/a	https://harvestchoice.org/data/area_nonf	12 Feb 2018	48
Banana	Banana and plantain production in 2005 (metric tonne)	static	n/a	https://harvestchoice.org/data/bapl_p	8 Feb 2018	49
Barley	Barley production in 2005 (metric tonne)	static	n/a	https://harvestchoice.org/data/barl_p	8 Feb 2018	50
Bean	Bean production in 2005 (metric tonne)	static	n/a	https://harvestchoice.org/data/bean_p	8 Feb 2018	51
Cassava	Cassava production in 2005 (metric tonne)	static	n/a	https://harvestchoice.org/data/cass_p	8 Feb 2018	52
Cereal	Cereal production in 2005 (metric tonne)	static	n/a	https://harvestchoice.org/data/cere_p	8 Feb 2018	53
Chickpea	Chickpea production in 2005 (metric tonne)	static	n/a	https://harvestchoice.org/data/chic_p	8 Feb 2018	54
Coconut	Coconut production in 2005 (metric tonne)	static	n/a	https://harvestchoice.org/data/cnut_p	8 Feb 2018	56
Coffee	Coffee production in 2005 (metric tonne)	static	n/a	https://harvestchoice.org/data/coff_p	8 Feb 2018	57
Cowpea	Cowpea production in 2005 (metric tonne)	static	n/a	https://harvestchoice.org/data/cowp_p	8 Feb 2018	58
Groundnut	Groundnut production in 2005 (metric tonne)	static	n/a	https://harvestchoice.org/data/grou_p	8 Feb 2018	59
Lentil	Lentil production in 2005 (metric tonne)	static	n/a	https://harvestchoice.org/data/lent_p	12 Feb 2018	60
Millet	Millet production in 2005 (metric tonne)	static	n/a	https://harvestchoice.org/data/mill_p	12 Feb 2018	61
Other fibres	Other fibre crop production in 2005 (metric tonne)	static	n/a	https://harvestchoice.org/data/ofib_p	12 Feb 2018	63
Other oils	Other oil crop production in 2005 (metric tonne)	static	n/a	https://harvestchoice.org/data/ooil_p	12 Feb 2018	64
Other root crops	Other roots and tubers crop production in 2005 (metric tonne)	static	n/a	https://harvestchoice.org/data/orts_p	12 Feb 2018	66
Palmoil	Palm oil production in 2005 (metric tonne)	static	n/a	https://harvestchoice.org/data/oilp_p	12 Feb 2018	67
Pigeonpea	Pigeonpea production in 2005 (metric tonne)	static	n/a	https://harvestchoice.org/data/pige_p	12 Feb 2018	68
Pulses	Pulses production in 2005 (metric tonne)	static	n/a	https://harvestchoice.org/data/puls_p	12 Feb 2018	70
Rapeseed	Rapeseed production in 2005 (metric tonne)	static	n/a	https://harvestchoice.org/data/rape_p	12 Feb 2018	71

Sesame	Sesame production in 2005 (metric tonne)	static	n/a	https://harvestchoice.org/data/sesa_p	12 Feb 2018	72
Sorghum	Sorghum production in 2005 (metric tonne)	static	n/a	https://harvestchoice.org/data/sorg_p	22 Apr 2018	73
Soybean	Soybean production in 2005 (metric tonne)	static	n/a	https://harvestchoice.org/data/soyb_p	12 Feb 2018	74
Sunflower	Sunflower production in 2005 (metric tonne)	static	n/a	https://harvestchoice.org/data/sunf_p	12 Feb 2018	75
Temperate fruit	Temperate fruits production in 2005 (metric tonne)	static	n/a	https://harvestchoice.org/data/temf_p	12 Feb 2018	77
Tea	Tea production in 20005 (metric tonne)	static	n/a	https://harvestchoice.org/data/teas_p	12 Feb 2018	78
Tobacco	Tobacco production in 2005 (metric tonne)	static	n/a	https://harvestchoice.org/data/toba_p	12 Feb 2018	79
Tropical fruit	Tropical fruits production in 2005 (metric tonne)	static	n/a	https://harvestchoice.org/data/trof_p	12 Feb 2018	80
Vegetables	Vegetables production in 2005 (metric tonne)	static	n/a	https://harvestchoice.org/data/vege_p	12 Feb 2018	81
Wheat	Wheat production in 2005 (metric tonne)	static	n/a	https://harvestchoice.org/data/whea_p	12 Feb 2018	82
Yams	Yams production in 2005 (metric tonne)	static	n/a	https://harvestchoice.org/data/yams_p	12 Feb 2018	83
Irrigated area	Areas equipped for irrigation	static	n/a	http://www.fao.org/nr/water/aquastat/irrigationmap/index10.stm	28 Aug 2018	84
Harvested area	Harvested area of all crops in 2005	static	n/a	http://harvestchoice.org/data/area_crop	27 Feb 2018	85
Grazing prod.	Grazing production for bovines in 2000 (kg/ha)	static	n/a	https://harvestchoice.org/data/bv_graz	30 May 2018	86
Stover prod.	Stover production for bovines in 2000 (kg/ha)	static	n/a	https://harvestchoice.org/data/bv_stov	30 May 2018	87
Cattle	Cattle density in 2005 (head/km^2)	static	n/a	https://harvestchoice.org/data/ad05_catt	30 May 2018	88
Sheep	Sheep density in 2005 (head/km^2)	static	n/a	https://harvestchoice.org/data/ad05_shee	30 May 2018	89
Goats	Goat density in 2005 (head/km^2)	static	n/a	https://harvestchoice.org/data/ad05_goat	30 May 2018	90
Pigs	Pig population in 2005 (head)	static	n/a	https://harvestchoice.org/data/an05_pig	30 May 2018	91
Chickens	Poultry density in 2005 (head/km^2)	static	n/a	https://harvestchoice.org/data/ad05_chic	30 May 2018	92
Livestock density	Livestock density in 2005 (LU/km^2)	static	n/a	https://harvestchoice.org/data/ad05_lu	30 May 2018	93
**Other land cover variables**						

Evergreen broadleaf percentage	Proportional cover of evergreen broadleaf forest (>60% land covered with broadleaf vegetation of height >2m and canopy never without green foliage)	annual	0, 1, 2, 3 years	https://modis.gsfc.nasa.gov/data/dataprod/mod12.php	30 Jul 2018	43
Mixed forest percentage	Proportional cover of mixed forest (>60% land covered with vegetation of height >2m and mosaic of the four forest types)	annual	0, 1, 2, 3 years	https://modis.gsfc.nasa.gov/data/dataprod/mod12.php	30 Jul 2018	43
Closed shrubland percentage	Proportional cover of closed shrublands (woody vegetation <2m tall with canopy cover >60% of area)	annual	0, 1, 2, 3 years	https://modis.gsfc.nasa.gov/data/dataprod/mod12.php	23 March 2018	43
Open shrubland percentage	Proportional cover of open shrublands (vegetation <2m tall and shrub canopy cover >60% of area)	annual	0, 1, 2, 3 years	https://modis.gsfc.nasa.gov/data/dataprod/mod12.php	23 March 2018	43
Woody savanna percentage	Proportional cover of woody savanna (trees 30-60% and understory vegetation)	annual	0, 1, 2, 3 years	https://modis.gsfc.nasa.gov/data/dataprod/mod12.php	23 March 2018	43
Savanna percentage	Proportional cover of savanna (trees 10-30% and understory vegetation)	annual	0, 1, 2, 3 years	https://modis.gsfc.nasa.gov/data/dataprod/mod12.php	30 Jul 2018	43
Grassland percentage	Proportional cover of grasslands (herbaceous cover with trees/shrubs <10%)	annual	0, 1, 2, 3 years	https://modis.gsfc.nasa.gov/data/dataprod/mod12.php	23 March 2018	43
Permanent wetland percentage	Proportional cover of permanent wetlands (a permanent mixture of water and vegetation over extensive areas)	annual	0, 1, 2, 3 years	https://modis.gsfc.nasa.gov/data/dataprod/mod12.php	23 March 2018	43
Barren and sparsely populated area percentage	Proportional cover of barren and sparsely populated areas (land with exposed soil, sand or rocks, with <10% vegetation cover at any time)	annual	0, 1, 2, 3 years	https://modis.gsfc.nasa.gov/data/dataprod/mod12.php	2 Aug 2018	43
**Other variables**						
Population density	Human population size (No. persons/pixel)	annual	0, 1, 2, 3 years	https://www.worldpop.org/geodata/listing?id=17	n/a	94
Drainage class	Classification for the rate at which water infiltrates into the soil.	n/a	n/a	http://data2.isric.org/geonetwork/srv/api/records/953d0964-6746-489a-a8d1-f188595516a9	9 Nov 2018	95
Soil moisture	Moisture content of a soil (%).	n/a	n/a	https://smap.jpl.nasa.gov/data/	1 Dec 2018	96
Bedrock	Depth at which bedrock occurs (cm)	n/a	n/a	https://files.isric.org/soilgrids/data/recent/	1 Dec 2018	97
Flow accumulation	Based on the digital elevation model a map on flow accumulation was created.	n/a	n/a	https://hydrosheds.org/	26 July 2018	98
Slope	Slope of the land (°)	n/a	n/a	https://cgiarcsi.community/data/srtm-90m-digital-elevation-database-v4-1/	23 March 2018	99
Soil depth	Depth of the soil layer (cm)	n/a	n/a	n/a	n/a	100
Rainfall erosivity factor	Factor that indicates the kinetic energy of raindrop's impact and the rate of associated runoff.	n/a	n/a	https://esdac.jrc.ec.europa.eu/content/global-rainfall-erosivity	4 Sep 2018	101
Slope-length factor	Factor that describes the effect of slope steepness and the impact of slope length.	n/a	n/a	n/a	n/a	42

Erosion	Total detachment and removal of soil material by water (t/ha/yr).	n/a	n/a	n/a	n/a	42
Cation exchange capacity	Cation exchange capacity of a soil is a measure for the amount of cations that can retain on soil particle surfaces ($cmol_c$/kg)	n/a	n/a	https://files.isric.org/soilgrids/data/recent/	21 Feb 2018	97
Clay content	Percent of clay particles (<2μm) in the soil (%).	n/a	n/a	https://files.isric.org/soilgrids/data/recent/	21 Feb 2018	97
Soil organic carbon	Organic carbon content in the soil (g/kg)	n/a	n/a	https://files.isric.org/soilgrids/data/recent/	21 Feb 2018	97
Soil pH	Soil pH is a measure of acidity or alkalinity of a soil.	n/a	n/a	https://files.isric.org/soilgrids/data/recent/	6 Feb 2018	97
GUF	Binary map of urban areas in 2011 (areas featuring man-made building structures with a vertical component)	static	no	https://www.dlr.de/eoc/en/desktop default.aspx/tabid-9628/16557_read-40454/	7 Feb 2017	102
**Climatic variables**						
Solar rad. †	Solar radiation (kJ/m²/day)	monthly	n/a	http://worldclim.org/version2	4 Sep 2018	103
Wind speed†	Long-term (1970-2000) average wind speed (m/s)	monthly	n/a	http://worldclim.org/version2	16 Jan 2018	103
Relative humidity	Average relative humidity (ratio of the partial pressure of water vapour to the equilibrium vapour pressure of water) between 2015 and 2018 (%)	static	n/a	https://developers.google.com/earth-engine/datasets/catalog/NOAA_GFS0P25	3 Dec 2018	104
Vegetation index max†	Maximum enhanced vegetation index is a measure of greenness reflectance of the land surface	annual monthly	0, 1, 2, 3 years	https://lpdaac.usgs.gov/products/mcd43d6*2-4*v006/	17 Sep 2018	105
Vegetation index mean†	Mean enhanced vegetation index is a measure of greenness reflectance of the land surface	annual monthly	0, 1, 2, 3 years	https://lpdaac.usgs.gov/products/mcd43d6*2-4*v006/	17 Sep 2018	105
Vegetation index min†	Minimum enhanced vegetation index is a measure of greenness reflectance of the land surface	annual monthly	0, 1, 2, 3 years	https://lpdaac.usgs.gov/products/mcd43d6*2-4*v006/	17 Sep 2018	105
Land surface temp. day max†	Maximum land surface daytime temperature (°C) gap-filled from the source.	annual monthly	0, 1, 2, 3 years	https://lpdaac.usgs.gov/products/mod11a2v006/	9 Oct 2018	106
Land surface temp. day mean†	Mean land surface daytime temperature (°C) gap-filled from the source.	annual monthly	0, 1, 2, 3 years	https://lpdaac.usgs.gov/products/mod11a2v006/	9 Oct 2018	106
Land surface temp. day min†	Minimum land surface daytime temperature (°C) gap-filled from the source.	annual monthly	0, 1, 2, 3 years	https://lpdaac.usgs.gov/products/mod11a2v006/	9 Oct 2018	106
Land surface temp. diurnal diff max†	Maximum difference between corresponding surface daytime temperature and surface night-time temperature images (°C)	annual monthly	0, 1, 2, 3 years	https://lpdaac.usgs.gov/products/mod11a2v006/	4 Oct 2018	106
Land surface temp. diurnal diff mean†	Mean difference between corresponding surface daytime temperature and surface night-time temperature images (°C)	annual monthly	0, 1, 2, 3 years	https://lpdaac.usgs.gov/products/mod11a2v006/	4 Oct 2018	106
Land surface temp. diurnal diff min†	Minimum difference between corresponding surface daytime temperature and surface night-time temperature images (°C)	annual monthly	0, 1, 2, 3 years	https://lpdaac.usgs.gov/products/mod11a2v006/	4 Oct 2018	106

Land surface temp. night max†	Maximum land surface night-time temperature (°C)	annual monthly	0, 1, 2, 3 years	https://lpdaac.usgs.gov/products/mod11a2v006/	5 Oct 2018	106
Land surface temp. night mean†	Mean land surface night-time temperature (°C)	annual monthly	0, 1, 2, 3 years	https://lpdaac.usgs.gov/products/mod11a2v006/	5 Oct 2018	106
Land surface temp. night min†	Minimum land surface night-time temperature (°C)	annual monthly	0, 1, 2, 3 years	https://lpdaac.usgs.gov/products/mod11a2v006/	5 Oct 2018	106
Rainfall†	Total precipitation (mm)	annual monthly	0, 1, 2, 3 years	http://chg.geog.ucsb.edu/data/chirps/#_Data	27 Nov 2017	107
Rainfall Intensity†	Average precipitation intensity (total precipitation/No. precipitation days) (mm)	annual monthly	0, 1, 2, 3 years	http://chg.geog.ucsb.edu/data/chirps/#_Data	11 Dec 2018	107
Bare surface moisture max†	Maximum values for a measure of moisture on bare surfaces (TCB, variation in soil background reflectance)	annual monthly	0, 1, 2, 3 years	https://lpdaac.usgs.gov/products/mcd43d6*2-4*v006/	6 Dec 2018	108
Bare surface moisture mean†	Mean values for a measure of moisture on bare surfaces (TCB, variation in soil background reflectance)	annual monthly	0, 1, 2, 3 years	https://lpdaac.usgs.gov/products/mcd43d6*2-4*v006/	6 Dec 2018	108
Bare surface moisture min†	Minimum values for a measure of moisture on bare surfaces (TCB, variation in soil background reflectance)	annual monthly	0, 1, 2, 3 years	https://lpdaac.usgs.gov/products/mcd43d6*2-4*v006/	6 Dec 2018	108
Surface wetness max†	Maximum values for a measure of surface moisture (TCW, variation in the vigour of green vegetation)	annual monthly	0, 1, 2, 3 years	https://lpdaac.usgs.gov/products/mcd43d6*2-4*v006/	3 Oct 2018	108
Surface wetness mean†	Mean values for a measure of surface moisture (TCW, variation in the vigour of green vegetation)	annual monthly	0, 1, 2, 3 years	https://lpdaac.usgs.gov/products/mcd43d6*2-4*v006/	3 Oct 2018	108
Surface wetness min†	Minimum values for a measure of surface moisture (TCW, variation in the vigour of green vegetation)	annual monthly	0, 1, 2, 3 years	https://lpdaac.usgs.gov/products/mcd43d6*2-4*v006/	3 Oct 2018	108
Potential evapotranspiration max	Max. potential evapotranspiration (water vapour flux under ideal conditions) between 1950 and 2000 (mm)	static	n/a	https://cgiarcsi.community/data/global-aridity-and-pet-database/	5 Feb 2015	109
Potential evapotranspiration mean	Mean potential evapotranspiration (water vapour flux under ideal conditions) between 1950 and 2000 (mm)	static	n/a	https://cgiarcsi.community/data/global-aridity-and-pet-database/	5 Feb 2015	109
Potential evapotranspiration min	Min. potential evapotranspiration (water vapour flux under ideal conditions) between 1950 and 2000 (mm)	static	n/a	https://cgiarcsi.community/data/global-aridity-and-pet-database/	5 Feb 2015	109
Potential evapotranspiration st.dev.	Standard deviation of the potential evapotranspiration (water vapour flux under ideal conditions) between 1950 and 2000 (mm)	static	n/a	https://cgiarcsi.community/data/global-aridity-and-pet-database/	5 Feb 2015	109
Elevation	Elevation measured using the hydrologically conditioned Digital Elevation Model (m)	n/a	n/a	https://hydrosheds.org/	27 March 2018	98
Distance water	Distance to water, including rivers, surface waters and oceans (m)	n/a	n/a	https://hydrosheds.org/	5 Nov 2015	98

† Conducted a principal component analysis on variables for each month and selected the top three principal components.

Table S4. The location in each country for which ICE relationships between predicted *Vgsc*-995F frequencies and ITN coverage were calculated.

Country	Latitude (°E)	Longitude (°N)
Burkina Faso	11.40145	-4.41939
Benin	9.350000	2.616670
Cameroon	5.969440	11.227220
Equatorial Guinea	1.91847	10.63520
Ethiopia	8.23333	37.58333
Sudan	14.14377	33.55163
Uganda	0.85707	33.9208
Kenya	-0.08262	34.77468
DRC	-3.5156740	23.59525
